# Ancient Protein Partners Take on Additional Roles in Multicellular Animals

**DOI:** 10.1371/journal.pbio.0040266

**Published:** 2006-07-04

**Authors:** Richard Robinson

Over the long course of life's history, the appearance of a new function in an organism may be accompanied by a new protein. But, more often, the work is done by an old one that adds a new role to its repertoire. Such proteins are likely to be found in a wide variety of organisms, reflecting their ancient lineage and continuing relevance. Proteins never act in isolation, of course; instead, they bind to one or more others to carry out their tasks. And so, if one member of a protein pair has taken on a new function, it's a good bet the other may have done so as well. In a new study, Vlad Seitan, Tom Strachan, and colleagues show that two proteins, whose interactions in yeast help chromosomes divide, have counterparts in a full range of other organisms, including humans. And true to prediction, the proteins don't just continue to play their old roles—in animals, they also appear to help guide multicellular development.

The focus of the study is a pair of yeast proteins, Scc2 and Scc4. Bound together, they load the protein complex cohesin onto chromosomes to link together sister chromatids, ensuring proper separation in mitosis. Scc2 has orthologs—proteins with similar structure sharing a common ancestor—in both fruit flies and humans, known respectively as Nipped-B and delangin. But, until very recently, orthologs of Scc4 have not been found outside of a few fungal species.

The authors set out to find binding partners for Nipped-B and delangin. Using Nipped-B as the bait, they snagged the protein product of the fly gene CG4203. The human counterpart of this protein, called KIAA0892, bound to delangin. Because both *CG4203* and KIAA0892 are related to a nematode protein called MAU-2, the authors dubbed them fly and human MAU-2. Each of these was about the same size as Scc4 and, using specialized bioinformatics approaches, they confirmed that the sequences of all three were related. Thus, Scc2 is to Scc4 as Nipped-B is to fly MAU-2, and delangin is to human MAU-2.

Up to this point, the only demonstrated functional similarity between Scc4 and the MAU-2s was their ability to bind their respective partners. To test whether human MAU-2 had a similar role in linking sister chromatids, the authors used RNA interference to diminish MAU-2 expression. When the level of MAU-2 was low, less cohesin was loaded onto the chromosomes, and sister chromatids prematurely separated, just as in yeast.

The nematode version of MAU-2 was originally identified as having a role in guiding cell movements and growth of axons during development. Did it also play a part in chromatid cohesion in the worm? Once again, RNA interference showed it did. Finally, if MAU-2 has a developmental role in the worm, what about in other organisms? When the authors used antisense to reduce MAU-2 in the frog, early development was delayed and the embryo displayed multiple defects. Reduction of frog delangin caused similar defects, indicating the two likely pair in this organism as well.

These findings shed light on the mechanics of chromatid cohesion, and will be useful for further elucidating the complex means by which chromatids remain together and then separate during mitosis. They also indicate that both subunits take part in shaping development. How they do so is not yet clear, but the role of the pair in controlling chromosome structure suggests they may help modify chromatin outside of the events of mitosis. Further study of this activity will likely help illuminate the pathologic mechanism of a rare human developmental disorder, Cornelia de Lange syndrome, which can be caused by a mutation in the delangin gene and which is characterized by low birth weight, slow growth, and multiple physical abnormalities.

## 

**Figure:**
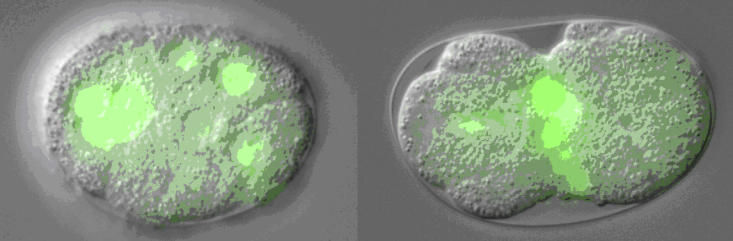
Simultaneous knockdown of MAU-2 and SCC-3 (left panel) or of PQN-85 and SCC-3 (right panel) consistently produces severe chromosome segregation defects in early worm embryos where the chromosomes do not appear to move to either pole.

